# State-Level Extreme-Risk Protection Order Policies, Mental Health, and Gun Carrying: Demographic and Racial Disparities in U.S. Youth

**DOI:** 10.1007/s11121-026-01912-4

**Published:** 2026-04-11

**Authors:** Xiang Gao

**Affiliations:** 1 Department of Public Health Education, School of Health and Human Sciences, University of North Carolina Greensboro, 437E Coleman Building, 1408 Walker Avenue, Greensboro, NC 27402 USA; 2 Carolina Asia Center, UNC Chapel Hill, Chapel Hill, NC USA; 3 Carolina Center for Population Aging and Health, UNC Chapel Hill, Chapel Hill, NC USA

**Keywords:** Extreme-Risk Protection Orders (ERPOs), Gun carrying, High school students, Demographic and racial disparities, Youth Risk Behavior Surveillance System (YRBS)

## Abstract

Gun carrying among U.S. high school students increases risks for violence and injury. Poor mental health is linked to carrying guns, but less is known about how this link varies across demographic and racialized groups. Extreme-risk protection orders (ERPOs) allow temporary removal of firearms from individuals at high risk of harming themselves or others, including during a mental health crisis. Their potential to reduce disparities in youth gun carrying is not well studied. This study assessed whether ERPOs modify the association between poor mental health and gun carrying in high school students overall and subgroups. Data came from the 2023 pooled state Youth Risk Behavior Surveillance System. Gun carrying was the outcome, poor mental health was the predictor, and ERPOs were the moderator, categorized as EROP (less restrictive), Ex parte (moderately restrictive), and Ex parte expanded (more restrictive). Sex, grade, and race were included. Multilevel mixed-effects logistic regression models tested interactions between ERPOs and poor mental health. Demographic and racialized differences in gun carrying were identified. Males had higher odds of carrying guns than females. Poor mental health was associated with higher odds of gun carrying overall and in subgroups, with stronger associations among males, 12th graders, African American students, and Hispanic students. Across the sample and subgroups, ERPOs were linked to reduced gun carrying among students reporting poor mental health. Findings suggest that ERPOs may lower the risk of gun carrying linked to poor mental health and may also reduce demographic and racialized disparities in youth firearm harm.

## Introduction

Gun carrying among adolescents is a major public health concern because it increases the risk of violence, injury, and death. National surveillance data from the Youth Risk Behavior Surveillance System indicate that about 3–5% of U.S. high school students reported carrying a gun in 2022, with clear differences across demographic groups (Brener, 2024; Centers for Disease Control & Prevention, 2023b). Boys report gun carrying at rates three to four times higher than girls, and rates rise through high school grades, with the highest levels observed among 12th graders (Brener, 2024). Racial and ethnic differences also remain a serious concern: Black and Hispanic youth carry guns at higher rates than White youth, a pattern linked to concentrated disadvantage, neighborhood violence exposure, and unequal access to supportive community resources (Oliphant et al., 2019; Sokol et al., 2022). Mental health plays an important role as well. Studies show that youth experiencing depressive symptoms, psychological distress, or suicidal thoughts have higher odds of carrying a gun, often as a response to perceived threats, low safety, or emotional strain (Abba-Aji et al., 2024; Ranney et al., 2019).

Extreme-risk protection orders (ERPOs) policies are civil legal frameworks that allow the temporary removal of firearms from individuals who pose a risk of harming themselves or others, with variation in policy design and implementation across states (Wintemute et al., 2019; Zeoli et al., 2025). Unlike criminal justice approaches, ERPOs focus only on risk and do not create a permanent record, which makes them distinct from conventional firearm prohibitions (Zeoli et al., 2025). States vary in the level of restrictiveness, but ERPOs generally involve a petition process initiated by family members, law enforcement, or other designated parties, followed by a court order that restricts access to guns during periods of heightened risk (Zeoli et al., 2025). Evidence suggests that ERPOs reduce firearm suicide and may also decrease the likelihood of firearm-related assault by lowering immediate access to guns during crises marked by distress, threat perceptions, or escalating conflict (Rowhani-Rahbar et al., 2020; Zeoli et al., 2025). ERPOs may reduce gun carrying among youth with mental health challenges through several connected pathways. First, removing firearms from a household or social network reduces opportunities for adolescents to access and carry firearms, which is a key mechanism linking firearm availability to youth firearm behaviors (Carter et al., 2020; Zeoli et al., 2019). Second, the ERPOs process often involves contact with trained professionals, which can link families to supportive services and mental health resources. This additional support may help connect families to mental health services, which may reduce distress and related risk behaviors (Zeoli et al., 2025). Third, ERPOs can interrupt chain reactions that occur when youth exposed to violence or community-level instability receive mixed messages about safety, leading them to carry firearms to reduce perceived threats (Sweeten et al., 2013). Additionally, fundamental cause theory and structural perspectives on health inequities help explain why ERPOs may operate differently across demographic and racial groups (Bottiani et al., 2021; Phelan & Link, 2015). These frameworks argue that health inequities are driven by structural conditions that shape access to resources, exposure to risk, and responses to harm across populations (Bottiani et al., 2021; Phelan & Link, 2015). These structural conditions shape exposure to risk, access to resources, and responses to harm (Phelan & Link, 2015). For firearm behaviors, this means that youth from racialized groups – particularly Black and Hispanic adolescents – are more likely to live in areas where violence exposure, neighborhood disadvantage, and reduced access to supportive services elevate both distress and the likelihood of carrying a gun (Baiden et al., 2024).

Although research has documented demographic and racial differences in youth gun carrying and its association with mental health challenges, the evidence base remains limited in several important areas. Current studies show that psychological distress, depressive symptoms, and suicidal thoughts increase the likelihood of gun carrying (Chung et al., 2020; Yang, 2023), and that these patterns differ across racial and ethnic groups because of unequal exposure to violence, disadvantage, and limited access to supportive services (Carey & Coley, 2022; Simon, 2022). However, most research focuses on individual-level factors and does not examine policy mechanisms that may moderate the association between mental health problems and gun carrying. Evidence on ERPOs is growing, but existing studies primarily focus on suicide prevention among adults or evaluate changes in firearm violence at the population level (Swanson et al., 2017; Zeoli et al., 2024, 2025). Few studies examine whether ERPOs lower gun carrying among adolescents, despite the central role of firearm access in youth safety and the increased distress observed in recent national data. Importantly, no research has tested whether ERPOs moderate the strength of the mental health-gun carrying association differently across sex, grade, and racial or ethnic groups, even though ERPOs’ implementation and community conditions vary widely across the United States. To address this gap, the current study investigates whether variation in state-level ERPO policy environments moderates the association between poor mental health and gun carrying among U.S. high school students and examines whether this moderating effect differs across demographic and racial groups.

## Methods

### Sampling

This study used pooled data from the 2023 state-level Youth Risk Behavior Surveillance System (YRBS), a biennial survey conducted by the Centers for Disease Control and Prevention to monitor health behaviors among U.S. high school students in grades 9 through 12 (Brener, 2024). The YRBS uses a two-stage cluster sampling design to produce representative estimates for participating states. Students were eligible for inclusion if they completed the firearm carrying item and the mental health item used in this analysis. Survey weights provided by CDC were applied to account for sampling design, nonresponse, and demographic adjustments, allowing the results to represent the population of high school students in participating states (Brener, 2024). Such sampling strategies can be found in other studies (Gao et al., 2025; Gao, 2026).

### Participants

After excluding respondents with missing data on key variables (gun carrying, mental health status, sex, grade, race and ethnicity, and state identifiers), the final analytic sample included 87,469 students across the survey year.

### Ethics

Because this study used publicly available, de-identified data from YRBS and RAND’s Gun Policy in America, it did not involve human subjects research. As such, the analysis qualified for exemption from the Institutional Review Board (IRB).

### Measures

#### Gun Carrying (Dependent Variable)

Gun carrying was measured using the YRBS item asking whether students carried a gun during the past 12 months, excluding for hunting or sport (Brener, 2024). Responses were coded dichotomously (1 = any gun carrying in the past 12 months; 0 = none). This approach is consistent with prior studies using YRBS data, which focus on any firearm carrying as an indicator of risk behaviour due to its low base rate and public health relevance (Simon, 2022; Zeoli et al., 2019).

#### Poor Mental Health (Independent Variable)

Poor mental health was assessed during the YRBS item asking whether students experienced poor mental health during the past 30 days, defined as stress, sadness, or hopelessness that interfered with usual activities (Brener, 2024). Responses were coded dichotomously (1 = reported poor mental health during the past 30 days; 0 = none), consistent with prior YRBS-based studies that operationalize this measure as an indicator of recent psychological distress affecting daily functioning (Baiden et al., 2024; Ranney et al., 2019). YRBS mental health indicators have been widely used as predictors of firearm behaviors and other safety-related outcomes among adolescents (Baiden et al., 2024; Ranney et al., 2019).

#### Extreme Risk Protection Orders (Moderator)

ERPO status was assigned at the state level using publicly available policy classifications from RAND’s Gun Policy in America database (WANG & Smart, 2020). States were categorized based on the restrictiveness of their ERPO provisions at the time of data collection: 1) ERPO (less restrictive), 2) Ex parte (moderately restrictive), 3) Ex parte expanded (more restrictive), and 4) No ERPO law (WANG & Smart, 2020). These classifications reflect differences in who may petition, evidentiary standards, and the range of conditions under which firearms may be temporarily removed (Zeoli et al., 2025).

#### Demographic Variables (Covariates)

Demographic variables included sex (male, female), grade (9th-12th), and race/ethnicity (White, Black or African American, Hispanic/Latino, All other races). These variables were coded according to YRBS documentation and included as covariates due to well-documented differences in firearm behaviors and mental health across demographic groups (Brener, 2024; Oliphant et al., 2019; Yang, 2023).

Dichotomization of these variables was used to align with established YRBS analytic practices and to facilitate interpretation of associations with relatively low-prevalence outcomes such as gun carrying (Simon, 2022).

### Statistical Analysis

Descriptive statistics were first computed to summarize sample characteristics and the prevalence of gun carrying across demographic groups, mental health status, and ERPO categories. Differences between groups were examined using Rao-Scott chi-square tests, which adjust for complex survey features. Multilevel mixed-effects logistic regression models were fit to examine the association between poor mental health and gun carrying while accounting for the nesting of students within states. A random intercept for state was included to adjust for unobserved state-level differences, consistent with prior research using multilevel structures in firearm behavior analyses (Dong & Wilson, 2022). Fixed effects included poor mental health, ERPO category, sex, grade, and race/ethnicity. To test whether ERPOs moderated the association between poor mental health and gun carrying, interaction terms between ERPO category and poor mental health were added to the models. Separate subgroup models were estimated for sex, grade, and racial/ethnic groups to assess differential moderation effects. Analyses used a two-sided significance threshold of α = 0.05. All analyses were conducted using SAS 9.4 (SAS Institute Inc., Cary, NC).

## Results

The analytic sample included 87,369 high school students. Overall, 4.4% of students reported carrying a gun in the past 12 months. Gun carrying varied across demographic groups: males reported substantially higher levels than females (6.5% vs. 1.8%), and 12th graders reported higher levels than 9th graders. Differences also appeared across racial and ethnic groups, with Black and Hispanic students showing higher prevalence compared with White students. Students reporting poor mental health had higher levels of gun carrying than those without poor mental health. States without ERPO laws showed a higher prevalence of youth gun carrying than states with ERPOs, including those with ex parte and expanded provisions (Table 1).

**Table 1:** Characteristics of participants by gun carrying, 2021–2023 state pooled Youth Risk Behavior Surveillance survey sample

Variables	Total (N = 87,369)	No, gun carrying (N = 83,500)	Yes, gun carrying (N = 3869)	p-value
Year				0.462
2023	44.62 (1.20)	42.80 (1.16)	1.82 (0.10)	
2021	55.38 (1.20)	53.02 (1.14)	2.36 (0.11)	
Sex				< 0.001
Female	49.70 (0.49)	48.82 (0.50)	0.89 (0.07)	
Male	50.30 (0.49)	47.00 (0.43)	3.29 (0.14)	
Grade				0.018
9th	27.16 (1.52)	26.14 (1.47)	1.02 (0.07)	
10th	25.47 (0.89)	24.37 (0.87)	1.11 (0.08)	
11th	23.93 (1.13)	23.00 (1.11)	0.93 (0.06)	
12th	23.43 (1.26)	22.31 (1.21)	1.12 (0.09)	
Race				0.001
White	48.64 (1.50)	46.90 (1.46)	1.75 (0.11)	
Black or African American	14.54 (0.85)	13.80 (0.81)	0.74 (0.08)	
Hispanic/Latino	27.58 (1.42)	26.25 (1.37)	1.33 (0.10)	
All other races	9.24 (0.41)	8.88 (0.41)	0.36 (0.04)	
Poor mental health				0.031
No	69.57 (0.47)	66.59 (0.40)	2.97 (0.14)	
Yes	30.44 (0.47)	29.23 (0.45)	1.20 (0.08)	
Extreme-risk protection orders (ERPO)				< 0.001
No	82.59 (0.99)	78.93 (0.97)	3.65 (0.13)	
ERPO (Less restrictive)	7.37 (0.70)	7.18 (0.67)	0.18 (0.04)	
Ex parte	3.87 (0.36)	3.72 (0.35)	0.15 (0.01)	
Ex parte expanded (More restrictive)	6.17 (0.27)	5.98 (0.26)	0.19 (0.03)	

*Notes*. Complex survey features were employed. Values were expressed as % (standard error). The percentage (%) and standard errors were calculated based on the total sample (n = 87,369). p-value was calculated using Rao-Scott’s chi-square test. All other races included American Indian/Alaska Native Hawaiian/Other Pacific Islander

Multivariable logistic regression models indicated that males, 12th graders, and Black or Hispanic youth were each associated with increased odds of gun carrying after adjusting for covariates. 12th graders had higher odds than 9th graders (AOR = 1.29, p = 0.005). Black (AOR = 1.51, p < 0.001) and Hispanic (AOR = 1.38, p < 0.001) students had higher odds than White students. Poor mental health was independently associated with increased odds of gun carrying, with students reporting poor mental health showing 32% higher odds than those without poor mental health (AOR = 1.32, p < 0.001) (Table 2).

**Table 2:** Demographic variables associated with gun carrying, 2021–2023 state pooled Youth Risk Behavior Surveillance sample

Variables	Unadjusted OR (95% CI)	p-value	AOR (95% CI)*	p-value
Year				
2023	0.96 (0.85, 1.08)	0.463	0.92 (0.82, 1.03)	0.129
2021	Ref		Ref	
Sex				
Male	3.86 (3.20, 4.65)	< 0.001	4.14 (3.43, 4.99)	< 0.001
Female	Ref		Ref	
Grade				
12th	1.29 (1.09, 1.54)	0.004	1.29 (1.08, 1.54)	0.005
11th	1.03 (0.86, 1.24)	0.720	1.02 (0.85, 1.23)	0.825
10th	1.17 (0.96, 1.42)	0.116	1.16 (0.96, 1.42)	0.130
9th	Ref		Ref	
Race				
Black or African American	1.44 (1.14, 1.81)	0.002	1.51 (1.20, 1.90)	< 0.001
Hispanic/Latino	1.37 (1.13, 1.64)	0.001	1.38 (1.15, 1.66)	< 0.001
All other races	1.10 (0.82, 1.47)	0.538	1.22 (0.90, 1.65)	0.211
White	Ref		Ref	
Poor mental health				
Yes	1.09 (1.07, 1.09)	0.030	1.32 (1.13, 1.53)	< 0.001
No	Ref		Ref	
Extreme-risk protection orders (ERPO)				
ERPO	0.55 (0.42, 0.72)	< 0.001	0.54 (0.41, 0.71)	< 0.001
Ex parte	0.89 (0.78, 0.98)	0.025	0.81 (0.72, 0.92)	< 0.001
Ex parte expanded	0.69 (0.52, 0.92)	0.012	0.68 (0.51, 0.90)	0.007
No	Ref		Ref	

*Notes*. Complex survey features were employed. AOR = adjusted odds ratio. CI = confidence interval. The association between each demographic variable and sexual dating violence victimization was adjusted for other demographic factors. All other races included American Indian/Alaska Native, Asian, Native Hawaiian/Other Pacific Islander. All estimates were calculated with the total sample (n = 87,369)

Students living in states with ERPO laws showed significantly lower odds of gun carrying compared with those living in states without ERPOs. Less-restrictive ERPO laws were associated with a 46% reduction in odds (AOR = 0.54, p < 0.001). Ex parte and expanded ex parte laws were also associated with reduced odds of gun carrying (AOR = 0.81, p < 0.001, and AOR = 0.68, p = 0.007) (Table 3).

**Table 3:** Prevalence of gun carrying by poor mental health among overall high school students and subgroups, 2021–2023 state pooled Youth Risk Behavior Surveillance sample

	Total	Gun carrying	
		No poor mental health	Poor mental health
Overall	3869	71.19 (67.67, 74.71)	28.81 (25.29, 32.33)
Subgroup			
Male	2932	75.96 (72.20, 29.72)	24.04 (20.28, 27.80)
12th grade	829	71.06 (62.54, 79.59)	28.94 (20.41, 37.46)
Black or African American	612	80.16 (72.84, 87.51)	19.82 (12.49, 27.16)
Hispanic/Latino	952	72.44 (65.99, 78.89)	27.56 (21.11, 34.01)
ERPO	110	64.35 (52.85, 75.85)	35.65 (24.15, 47.15)
Ex parte	1595	65.44 (62.43, 68.45)	34.56 (31.55, 37.57)
Ex parte expanded	69	66.69 (55.25, 78.13)	33.31 (21.87, 44.74)

*Notes*. Values were expressed as % (95% Confidence Interval) based on the total sample (n = 87,369)

In the full sample, less-restrictive ERPO laws significantly reduced the strength of the association between poor mental health and gun carrying (AOR = 0.47, p = 0.023), and expanded ex parte provisions also reduced the strength of the association (AOR = 0.56, p = 0.047). Although interaction effects for standard ex parte laws were not statistically significant, the direction of the estimates suggested a protective trend. In the subgroup analyses, among males, poor mental health remained significantly associated with gun carrying (AOR = 1.44, p < 0.001), and the moderating effect of less-restrictive ERPOs was similar to that observed in the full sample (AOR = 0.46, p = 0.034). Among 12th graders, poor mental health remained significantly associated with gun carrying (AOR = 1.39, p < 0.001), though interaction effects were not statistically significant. For Black students, poor mental health increased the odds of gun carrying (AOR = 1.33, p = 0.044), and less-restrictive ERPOs significantly reduced this relationship (AOR = 0.43, p = 0.040). For Hispanic students, poor mental health also showed a significant association with gun carrying (AOR = 1.49, p < 0.001), with less-restrictive ERPOs again reducing the strength of this association (AOR = 0.36, p = 0.014) (Table 4).

**Table 4:** Multilevel logistic regression model results for interactions between EROP and poor mental health on gun carrying among overall and subgroup populations of high school students, 2021–2023 state pooled Youth Risk Behavior Surveillance sample

	Total	Model 1: bivariate			Model 2: controls			Model 3: controls and interactions											
								Main effect (poor mental health)			Interactions (ERPO × Poor mental health)								
											ERPO			Ex parte			Ex parte expanded		
		OR	S.E	p-value	AOR	S.E	p-value	AOR	S.E	p-value	AOR	S.E	p-value	AOR	S.E	p-value	AOR	S.E	p-value
Overall	87,369	1.08	0.04	0.033	1.50	0.04	< 0.001	1.37	0.05	< 0.001	0.47	0.36	0.023	0.81	0.25	0.406	0.56	0.29	0.047
Subgroup																			
Male	42,923	1.49	0.04	< 0.001	1.50	0.045	< 0.001	1.44	0.06	< 0.001	0.46	0.37	0.034	0.79	0.26	0.351	0.56	0.31	0.061
12th grade	16,984	1.10	0.08	0.003	1.39	0.08	< 0.001	1.12	0.12	0.309	0.75	0.42	0.495	0.64	0.28	0.109	0.52	0.43	0.126
Black or African American	13,619	1.01	0.10	0.009	1.41	0.10	< 0.001	1.33	0.14	0.044	0.43	0.41	0.040	0.66	0.31	0.177	0.95	0.51	0.920
Hispanic/Latino	19,509	1.19	0.07	0.013	1.57	0.07	< 0.001	1.49	0.12	< 0.001	0.36	0.41	0.014	0.86	0.25	0.566	0.85	0.35	0.633

*Notes*. Complex survey features were employed. OR = Odds Ratio. AOR = Adjusted odds ratio. ERPO = Extreme Risk Protection Order. CI = Confidence Interval. The association between poor mental health and gun carrying among overall high school students was adjusted for sex, grade, and race. Participants who did not report carrying were set as the reference group. All estimates were calculated with the total sample (n = 87,369). A more stringent criterion was used to interpret the statistical significance of multiple tests. 99% CIs were calculated with a significance level of 0.01 (*p < 0.01) to be considered statistically significant. The association between poor mental health and gun carrying in subgroup populations was adjusted for all demographic variables other than the stratified variable

## Discussion

This study is among the first studies to examine how poor mental health relates to gun carrying among U.S. high school students and whether state-level ERPO laws moderate this association across demographic and racial groups. The findings show that poor mental health is linked to higher odds of gun carrying, with notable differences across sex, grade, and racial and ethnic groups. Youth in states with ERPO laws, particularly less-restrictive provisions, had lower odds of gun carrying overall, and ERPOs reduced the strength of the association between poor mental health and gun carrying in the full sample and several subgroups.

The descriptive results show that 4.4% of U.S. high school students reported carrying a gun in the past year, with higher levels among males, older students, and Black and Hispanic youth. These patterns are consistent with national surveillance data from the YRBS, which similarly documents higher gun carrying among male adolescents and persistent racial and ethnic disparities shaped by unequal exposure to community violence and structural disadvantage (Baiden et al., 2024; Centers for Disease Control & Prevention, 2023a). The higher prevalence of gun carrying among adolescents reporting poor mental health aligns with prior work showing that psychological distress, sadness, and exposure to stressors are linked to increased firearm access and carrying among youth (Yang, 2023). The lower prevalence of gun carrying in states with ERPO laws suggests that policy environments may influence baseline firearm behavior among adolescents, consistent with emerging evidence that restrictive firearm policies can reduce risky firearm access and related harms (Zeoli et al., 2025). These descriptive patterns reinforce the importance of considering demographic, psychosocial, and policy contexts when examining drivers of adolescent firearm behavior.

Gun carrying is patterned along demographic and psychosocial lines. The higher odds of gun carrying among males, 12th graders, and Black and Hispanic students align with prior national analyses showing that older adolescents, boys, and youth from racialized groups are more likely to report carrying a firearm, often reflecting differences in exposure to neighborhood violence, safety concerns, and structural disadvantage (Baiden et al., 2024; Cunningham et al., 2015). The independent association between poor mental health and increased odds of gun carrying is also consistent with prior research demonstrating that adolescents experiencing sadness, hopelessness, or psychological distress have an elevated likelihood of engaging in firearm-related behaviors as a coping mechanism or response to perceived threats (Yang, 2023). These converging findings support the broader evidence that adolescent firearm behavior reflects an interplay between individual distress and structural contexts that shape perceived risk and access to protection.

The lower odds of gun carrying in states with ERPO laws suggest that limiting firearm access in high-risk contexts may help reduce unsafe firearm behaviors among adolescents; however, these states may also differ in other firearm policies, prevention efforts, and structural conditions that could contribute to the observed associations (Smart et al., 2023; Zeoli et al., 2025). Although most ERPO research focuses on adults, evidence consistently shows that ERPO implementation reduces firearm access during periods of elevated risk and is associated with preventing firearm suicides and disrupting threats of mass violence (Swanson et al., 2017; Wintemute et al., 2019). ERPOs that allow broader petitioning and more timely judicial action, features commonly seen in less-restrictive or ex parte provisions, have been shown to facilitate firearm recovery and reduce opportunities for impulsive firearm use (Frattaroli et al., 2020). These mechanisms offer plausible explanations for why less-restrictive ERPO laws in this study were associated with the strongest reductions in adolescent gun carrying, followed by ex parte and expanded ex parte provisions. These findings align with documented ERPO effects in adult populations and suggest that state policy environments may shape firearm behaviors even among youth, particularly by reducing access during periods of heightened distress or environmental threat.

The moderation findings indicate that ERPO laws can weaken the link between poor mental health and gun carrying, suggesting that reducing access to firearms during periods of elevated psychosocial risk may buffer against unsafe firearm behavior. The stronger protective associations observed for less-restrictive ERPO provisions are consistent with evidence showing that ERPOs are most effective when the petitioning process is accessible, timely, and widely used by eligible parties (Wintemute et al., 2019). The observation that expanded ex parte provisions also weakened the mental health–gun carrying association aligns with studies demonstrating that rapid, temporary firearm removal under ex parte orders can prevent firearm-related harm in moments of acute crisis (Swanson et al., 2017). Variation across subgroups, such as stronger moderation among males, Black youth, and Hispanic youth, may reflect differences in baseline firearm access, exposure to community violence, or perceived need for protection, patterns well documented in national studies on adolescent firearm behavior (Baiden et al., 2024). These subgroup differences are also consistent with fundamental cause theory and structural perspectives, which highlight how structural inequities shape both risk exposure and the benefits of preventive policy mechanisms. Taken together, the moderation results suggest that ERPOs may disrupt risk pathways linking distress to firearm carrying, with particularly meaningful effects for youth facing higher structural and contextual vulnerabilities.

The fundamental cause theory and structural perspectives help explain why the moderation effects varied across groups. Structural conditions like economic inequality, neighborhood violence exposure, and systemic disinvestment shape both mental health risks and patterns of firearm behaviors among racialized youth (Bottiani et al., 2021; Everytown for Gun Safety Support Fund, 2025). That ERPOs appeared to weaken the mental health-gun carrying link more strongly among some racial and ethnic groups may reflect how differential access to supportive services, community resources, and policy enforcement can influence behavioral responses to distress (Everytown Research & Policy, 2023; Smart et al., 2023; Swanson, 2020). Where policies reduce firearm access in contexts marked by concentrated disadvantage, the compounded risk that arises from intersecting social stressors may be attenuated, consistent with the theory’s emphasis on structural determinants (e.g., economic hardship, social disinvestment, neighborhood conditions) that significantly shape firearm violence risk (Buggs et al., 2022). Reducing firearm access through law can disrupt one part of this risk chain, thereby reducing the compounded risk that arises from intersecting social stressors (Buggs et al., 2022).

These findings reinforce that gun carrying among adolescents is shaped not only by individual mental health but also by the social and environmental conditions that influence safety, stress, and access to resources. Prior studies show that exposure to neighborhood violence, substance use, and safety concerns is linked to higher odds of firearm carrying, particularly in communities facing structural disadvantage (Baiden et al., 2024; Yang, 2023). Applying fundamental cause theory and structural perspectives underscores how these structural factors magnify risk for male, Black, and Hispanic youth, making efforts to address psychological distress and community conditions equally important. Strengthening early identification of mental health concerns in school and youth-serving settings, supported by clear referral pathways and staff training, aligns with national recommendations for promoting behavioral health (National Academies of Sciences Medicine Board on Children Youth Committee on Fostering Healthy Mental Behavioral Development Among Children Youth, 2019). At the community level, interventions such as violence interruption programs and trauma-informed school practices can help reduce perceived threats and reliance on firearms for protection (Branas et al., 2011). The finding that ERPO laws, especially less-restrictive versions, reduce overall gun carrying and weaken the association between mental health challenges and gun carrying suggests that policymakers can use ERPOs as a preventive tool when adolescents are at elevated risk. For this to be effective, frontline practitioners (e.g., clinicians, school psychologists, social workers, and crisis responders) must be aware of ERPO provisions, understand when they can petition, and receive training on how to use ERPOs safely and appropriately in collaboration with families (Zeoli et al., 2025). Strengthening community awareness campaigns, improving cross-sector communication, and ensuring culturally responsive implementation can help ERPOs operate more effectively in communities experiencing concentrated disadvantage. Taken together, these actions show how coordinated efforts across education, mental health, public health, and policy sectors can reduce the risks associated with youth firearm access and help mitigate inequities in firearm-related harm.

This study has several limitations. First, the cross-sectional YRBSS data do not allow the determination of whether poor mental health preceded gun carrying. Second, reliance on self-reported behaviors may introduce recall or social desirability bias, especially for sensitive items such as firearm carrying. Third, the mental health measure is broad and may not capture the full range of symptoms relevant to firearm behavior. Fourth, although ERPO classifications were based on a validated policy database, the analysis could not account for differences in implementation, enforcement, community awareness, or co-occurring state firearm policies across states (Zeoli et al., 2025). Fifth, unmeasured factors such as household firearm access, exposure to violence, and availability of mental health services may contribute to residual confounding. Finally, the survey includes only students attending school, potentially underestimating firearm involvement among youth who are chronically absent or disengaged from school.

Taken together, these findings underscore the interconnected roles of mental health, structural conditions, and policy environments in shaping adolescent gun carrying. The consistent association between psychological distress and firearm behavior highlights the need for stronger school- and community-based mental health supports, while the disparities observed across demographic and racial groups reflect broader inequities that influence safety and access to resources. The protective patterns associated with ERPO laws suggest that policy tools designed to limit firearm access during periods of heightened risk may help reduce youth gun carrying, particularly when implemented in ways that are accessible, culturally responsive, and integrated with local prevention efforts. By demonstrating how ERPOs can interrupt risk pathways for diverse groups of adolescents, this study offers evidence that coordinated approaches, linking mental health services, community violence prevention, and policy action, have the potential to reduce firearm-related harm and promote more equitable safety outcomes for young people.

## Data Availability

The data is publicly available at the CDC website. The code is available upon reasonable request based on the author’s discretion.
